# Supplementary material for: Investigation of the lncRNA THOR in Mice Highlights the Importance of Noncoding RNAs in Mammalian Male Reproduction
Source: Biomedicines. 2021 Jul 22;9(8):859. doi: 10.3390/biomedicines9080859 (PMC8389704; doi:10.3390/biomedicines9080859)
Supplement: Supplementary file 1 [file biomedicines-09-00859-s001.zip › Table S1.pdf]

Table S1: The primers of potential off-target sites (POTS) used in this study

		Potential Off Target Site	Number of mismatch	Position	PCR Primer
sgRNA1	OT1	tAaCCTgAAGGG<br>GTtTACGG-GGG	4	Chr19:<br>-33316	F:AAATGGATTGGGAGCCAGGG<br>R:ACCCCCTGGGTATTTGCCTA
	OT2	cAGCCTTAtGGG<br>tgCTACGG-GGG	4	Chr5:<br>+15099	F:TCCCTCTGCATACCCTCCTC<br>R:TATTAAATCCGCTCCCCCGC
	OT3	GAcCCTTctGGG<br>GgCTACGG-AGG	4	Chr6:<br>+128637	F:TGGCTGCCTGTGTAAGCAAGTC<br>R:CCTGCCTGGAGGTTTCACTGTT
sgRNA2	OT4	tgCACATCAaGC<br>ATtGGCAT-TGG	4	Chr9:<br>-36820	F:GGTGTGCTGCTCCCAATGCTAT<br>R:GGCCACTGTACTTGGTCCTCCT
	OT5	GAtACATCtgGC<br>ATgGGCAT-CGG	4	Chr12:<br>-122809	F:TGGCGCATCTACCAGATTTCA<br>R:ACCCAGAGTGAGGCACAGTA
	OT6	GACtgATgACaC<br>ATCGGCAT-GGG	4	Chr11:<br>-56255	F:GCACGCTGGGGACATAACTT<br>R:GCATCCCCAGGAGGAGTAGA
	OT7	GACAgAgCACcC<br>ATaGGCAT-AGG	4	Chr15:<br>+39770	F:CCAGAGGAAGACCAACCGTGTC<br>R:ACATTCCCAAGGCAGTGGAACT
	OT8	GACAgATgACtC<br>ATgGGCAT-CGG	4	Chr13:<br>+85077	F:GGAACTGCAGATGTCCCCTC<br>R:ATGATTGCTGCCACGGGTAA
	OT9	GACACAgCACGC<br>caaGGCAT-TGG	4	Chr15:<br>-149496	F:CTACCTGCTTTCCCGTGTGT<br>R:AAGGCTCACCCTGGACAATG
	OT10	GACACATCtgGg<br>ATtGGCAT-GGG	4	Chr16:<br>+147218	F:TTGGGCATCTTGCTCTCCAG<br>R:GCTGAACCCAGAGCTTCACT
