# Supplementary figures and images for: Investigation of the lncRNA THOR in Mice Highlights the Importance of Noncoding RNAs in Mammalian Male Reproduction
Source: Biomedicines. 2021 Jul 22;9(8):859. doi: 10.3390/biomedicines9080859 (PMC8389704; doi:10.3390/biomedicines9080859)

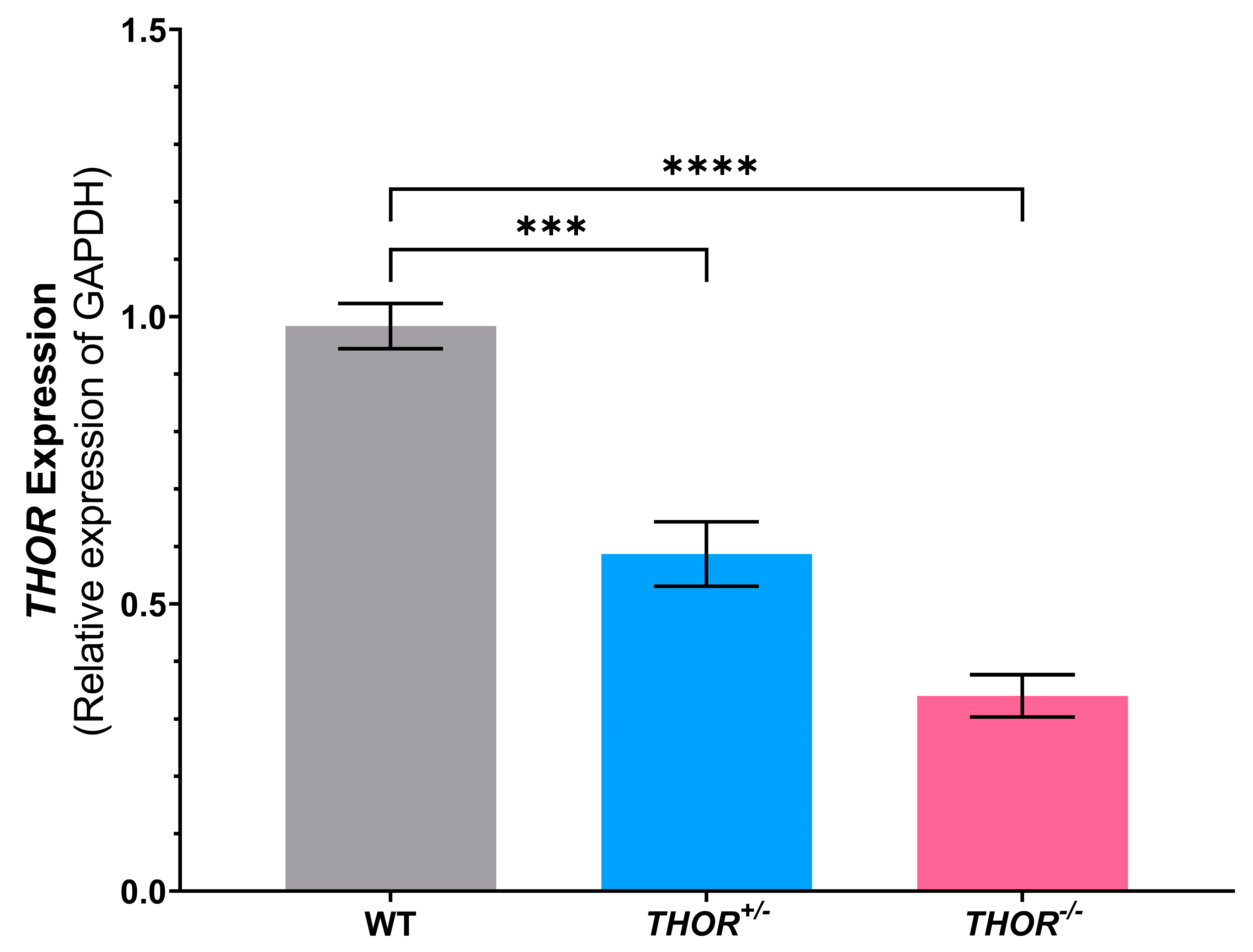

Supplement: Supplementary file 1 [file biomedicines-09-00859-s001.zip › Figure S2.tif]
